# Systemic absorption of triamcinolone acetonide is increased from intrasynovial versus extrasynovial sites and induces hyperglycemia, hyperinsulinemia, and suppression of the hypothalamic-pituitary-adrenal axis

**DOI:** 10.3389/fvets.2024.1388470

**Published:** 2024-05-15

**Authors:** Kimberly L. Hallowell, Katarzyna Dembek, Caitlyn R. Horne, Heather K. Knych, Kristen M. Messenger, Lauren V. Schnabel

**Affiliations:** ^1^Department of Clinical Sciences, College of Veterinary Medicine, North Carolina State University, Raleigh, NC, United States; ^2^K. L. Maddy Equine Analytical Pharmacology Laboratory, School of Veterinary Medicine, University of California-Davis, Davis, CA, United States; ^3^Department of Molecular Biomedical Sciences, College of Veterinary Medicine, North Carolina State University, Raleigh, NC, United States; ^4^Comparative Medicine Institute, North Carolina State University, Raleigh, NC, United States

**Keywords:** corticosteroids, triamcinolone, insulin, cortisol, sacroiliac, horse

## Abstract

Steroid-associated laminitis remains a major concern with use of corticosteroids in horses. Individual case factors such as joint pathology, pre-existing endocrinopathies, or corticosteroid type, dose, and timing influencing steroid-induced laminitis risk have not been investigated. This study aimed to determine if systemic absorption of triamcinolone acetonide (TA) varies between intrasynovial (antebrachiocarpal) and extrasynovial (sacroiliac) injection sites, and to determine the effects of TA absorption on glucose, insulin, cortisol, and adrenocorticotropic hormone (ACTH). Twenty adult horses were randomized into antebrachiocarpal or sacroiliac joint injection groups, and each horse received bilateral injections with a total dose of 18 mg triamcinolone. Blood was collected prior to injection and at 1, 2, 4, 6, 8, 10, 12, 16, 20, 24, 36, 48, 60, and 72 h post-injection. Peak TA absorption occurred at 8 h in both groups, and was significantly higher in the intrasynovial group compared to the extrasynovial group (1.397 ng/mL, 0.672 ng/mL, *p* < 0.05). Plasma TA levels were significantly higher in the intrasynovial group from 8 to 36 h post-injection (*p* < 0.05). There was no difference in glucose, insulin, cortisol, or ACTH between groups at any time point. Insulin and glucose were significantly increased from baseline at all timepoints from 10–72 h and 1–72 h post-injection, respectively. Horses with elevated baseline insulin values (>20 μU/mL) from both groups experienced a more marked hyperinsulinemia, reaching a mean peak insulin of 197.5 μU/mL as compared to 90.06 μU/mL in those with normal baseline insulin. Cortisol and ACTH were significantly decreased from baseline at timepoints from 4–72 h post-injection in both groups. This study is the first to evaluate drug absorption from the sacroiliac site and demonstrates that drug absorption varies between intrasynovial and extrasynovial injection sites. TA absorption causes metabolic derangements, most notably a marked hyperinsulinemia that is more severe in horses with elevated baseline insulin values. The influence of baseline endocrinopathies on response to corticosteroid administration as well as the effect of corticosteroid-induced metabolic derangements warrant further investigation as risk factors for corticosteroid-associated laminitis.

## 1 Introduction

Laminitis remains a major cause of animal suffering, economic loss, and emotional distress to owners and veterinarians. Laminitis is typically divided into three major categories: support-limb or mechanical laminitis, laminitis of inflammatory disease, and endocrinopathic laminitis. Of these three categories, endocrinopathic laminitis is the most common (1). Corticosteroid-associated laminitis is often discussed separately from the three primary categories, and evidence for the existence of corticosteroid-associated laminitis is lacking in the literature (2–7). Nevertheless, anecdotal reports and the authors’ clinical experience indicate that one-time administration of corticosteroids at an accepted “safe” dose can be sufficient to induce laminitis in certain patients. Recent literature has shown that treatment with corticosteroids such as triamcinolone acetonide (TA) can induce cortisol suppression, hyperglycemia, and hyperinsulinemia in equine patients. Hyperglycemia and hyperinsulinemia persist for up to 72 h, and cortisol suppression persists for 11 days with intra-articular triamcinolone and greater than 15 days with intramuscular triamcinolone (8–10). Hyperinsulinemia has become a focus of research into endocrinopathic laminitis and seems to be a driving risk factor for development of disease (11–13). In the most recent Recommendations for the Diagnosis and Management of Equine Metabolic Syndrome published by the Equine Endocrinology Group it is suggested that corticosteroid-induced hyperinsulinemia may increase risk of laminitis and that screening for insulin dysregulation prior to corticosteroid administration is warranted (14). Further research into the metabolic effects of steroid administration, particularly alterations in insulin production and sensitivity, may provide a better understanding of the pathophysiology of steroid-induced laminitis and in turn allow for prevention of disease.

In a recent study examining laminitis risk in horses receiving joint therapy with triamcinolone, extrasynovial sites such as the sacroiliac joint were specifically excluded (6), and previous studies have not separated horses by site of injection (3, 5, 6). The pharmacokinetic and pharmacodynamic properties of intrasynovial triamcinolone have been established, but only following administration in the antebrachiocarpal joint (8, 9, 15). The sacroiliac joint is unique when compared to other joint injection sites in that the injection is performed outside of a synovial compartment and without direct visualization of the joint itself (16–18). To our knowledge, no previous studies have evaluated the drug absorption properties of extrasynovial sites such as the sacroiliac joint.

To begin to understand the individual case factors that put horses at risk for steroid-induced laminitis, this study aimed to evaluate variations in triamcinolone absorption when injected at an extrasynovial site (sacroiliac joint) as compared to an intrasynovial site (antebrachiocarpal joint), and to determine the effects of systemic TA absorption on glucose, insulin, cortisol, and adrenocorticotrophic hormone (ACTH) levels. We hypothesized that there would be increased systemic triamcinolone absorption from extrasynovial sites as compared to intrasynovial sites, and that triamcinolone administration would result in increases in glucose and insulin and suppression of cortisol and ACTH concentrations.

## 2 Materials and methods

### 2.1 Animal use and welfare

The study protocol was approved by the North Carolina State University Institutional Animal Care and Use Committee (Protocol #22-115). Twenty horses (14 mares, 5 geldings, and 1 stallion) from the university teaching herd ranging in age from 2 to 20 years old were enrolled. The horses had a mean ± standard deviation (SD) body weight of 525 ± 42.3 kg. Breeds included 13 Quarter Horse/Paints, 4 Thoroughbreds, 1 Standardbred, 1 Tennessee Walking Horse, and 1 Warmblood. All horses were determined to be healthy based on physical examination and had no history of corticosteroid administration for at least six months prior to the study period and no history of laminitis. Horses were not tested for endocrinopathies prior to enrollment in the study. Horses were randomly assigned to intrasynovial (*n* = 10) or extrasynovial (*n* = 10) injection groups. The mean ± standard deviation (SD) age of the intrasynovial group was 11.8 ± 5.7 years and the mean ± SD age of the extrasynovial group was 11.4 ± 5.7 years. The mean ± SD body weight of the intrasynovial group was 516.9 ± 45.7 kg and the mean ± SD body weight of the extrasynovial group was 532.1 ± 37.1 kg. Horses entered the study in cohorts of 4 and were acclimated to the research stalls and a no grain diet for five days prior to injection. Horses were offered grass hay and water *ad libitum* throughout the study period. Horses were monitored for any signs of adverse reaction to injections including daily physical examinations with evaluation of injection sites, soundness at a walk, and digital pulses.

### 2.2 Articular injections

Horses in the intrasynovial group (IS) were sedated with detomidine and horses in the extrasynovial group (ES) with detomidine and butorphanol to facilitate articular injections. The hair over the intended injection sites was clipped and the skin was aseptically prepared using chlorhexidine solution and 70% isopropyl alcohol. Both antebrachiocarpal joints were flexed and injected via the standard cranial approach with 9 mg of triamcinolone acetonide and 50 mg of amikacin for a total volume of 1.1 mL each site and a total systemic dose of 18 mg triamcinolone acetonide and 100 mg amikacin. Both sacroiliac joints were injected via the previously described cranial ultrasound-guided approach with 9 mg of triamcinolone acetonide and 50 mg of amikacin diluted to 10 mL with 0.9% NaCl at each site for a total systemic dose of 18 mg triamcinolone acetonide and 100 mg amikacin (17). Injections were performed between 7:00 AM and 7:30 AM for all horses.

### 2.3 Sample collection and processing

Blood was obtained by direct jugular venipuncture prior to injection and at 1, 2, 4, 6, 8, 10, 12, 16, 20, 24, 36, 48, 60, and 72 h post-injection. Whole blood from the collection syringe was used for stall-side glucose testing. The remaining blood was separated into red top and EDTA blood tubes and stored on ice until centrifugation at 3000 × g for 20 min at 4C. Serum and plasma were then transferred to cryovials and stored at −80°C until analysis.

### 2.4 Quantification of TA, glucose, insulin, cortisol, and ACTH

Plasma triamcinolone levels were determined by liquid chromatography-mass spectrophotometry as previously described by Knych et al. (15). Whole blood glucose levels were determined at time of sampling using an Accu-Check point-of-care reader on the dog setting. Plasma insulin levels were determined by enzyme-linked immunosorbent assay (07 M-60102, MP Biomedicals, LLC, Solon, Ohio). Serum cortisol levels were determined by coated-tube radioimmunoassay (0722110-CF, MP Biomedicals, LLC, Solon, Ohio). Plasma ACTH concentration was determined using an automated chemiluminescent assay (CLIA; ACTH Immulite 2000 kit, Siemens Medical Solutions USA, Tarrytown, New York). All assays were performed at the North Carolina State University and have been previously published for use in equids (19–22).

### 2.5 Statistical analysis

Data were tested for normality by a D’Agostino-Pearson test. Normally distributed data were reported as mean and SD. Non-normally distributed data were reported as median and interquartile range. Repeated measures 2-way ANOVA test was used to compare triamcinolone, insulin, glucose, cortisol, and ACTH concentrations over time from baseline and between 2 groups (IS and ES) at each time point. Dunnett’s *post hoc* comparisons were made when relevant. Peak insulin concentration in horses with elevated and normal baseline insulin was compared with a *t*-test. Univariate logistic regression analysis was used to calculate the likelihood of peak insulin >100 uIU/mL in horses with elevated baseline insulin. The Hosmer and Lemeshow Goodness-of-Fit test indicated that the data fit the model (*p* = 0.74). Significance was set at *p* < 0.05. Statistical analysis was performed using Prism and IBM SPSS Statistical Software (SPSS and GraphPad Software, Inc., La Jolla, California).

## 3 Results

### 3.1 Physical examination parameters

No adverse reactions were noted and all horses had normal physical examination parameters throughout the study period. All horses tolerated repeated jugular venipuncture well with no changes in behavior or need for additional restraint.

### 3.2 Triamcinolone

**Figure 1:**
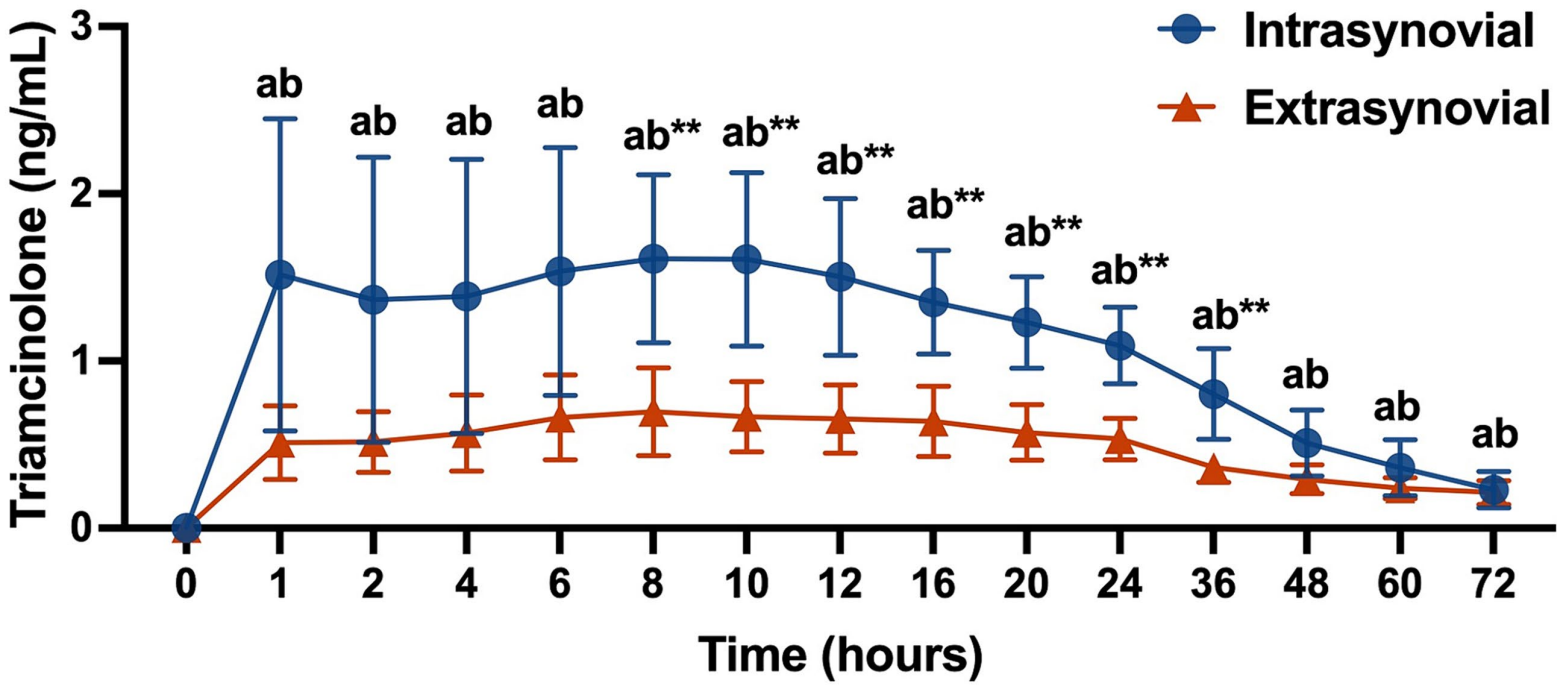
Plasma triamcinolone values (ng/mL) following intra- (*n* = 10) or extrasynovial (*n* = 10) injection with 18 mg triamcinolone acetonide. Data reported as mean and standard deviation for each group at all time points. Significant differences between groups are denoted by **(*p* < 0.01) and from baseline for the intrasynovial group by the letter a (*p* < 0.05) and for the extrasynovial group by the letter b (*p* < 0.05) as determined by 2-way repeated measures ANOVA.

Plasma triamcinolone levels for the IS versus ES group are displayed in Figure 1. TA levels were increased from baseline at all time points for both groups (*p* < 0.05). Plasma TA levels were greater in the IS group as compared to the ES group from 8 to 36 h post-injection (*p* < 0.01). Peak TA absorption occurred at 8 h post-injection in both groups and was significantly greater in the IS group than the ES group (1.61 ± 0.50 ng/mL vs. 0.70 ± 0.26 ng/mL, (*p* < 0.01)).

### 3.3 Glucose

**Figure 2:**
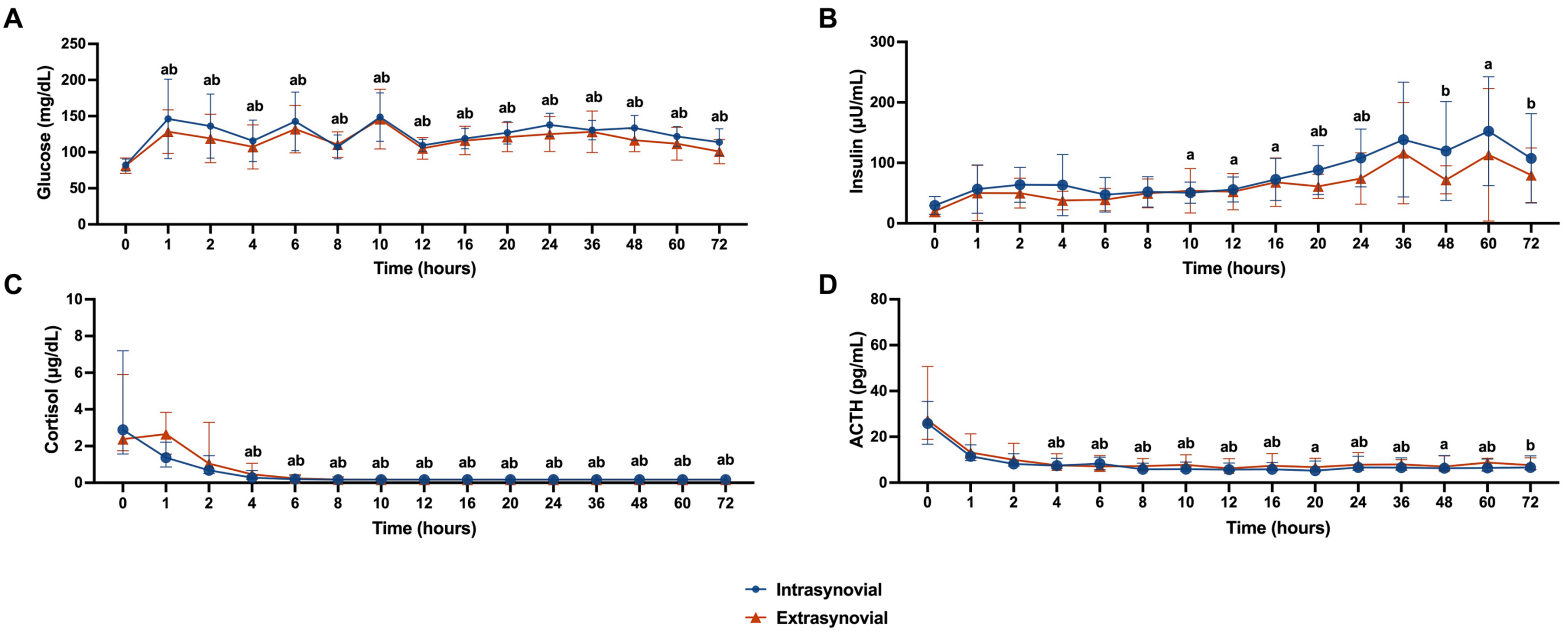
Metabolic parameters following intra- (*n* = 10) or extrasynovial (*n* = 10) injection with 18 mg triamcinolone acetonide. **(A)** Whole blood glucose (mg/dL), **(B)** plasma insulin (μU/mL), **(C)** serum cortisol (μg/dL), and **(D)** plasma ACTH (pg/mL). Normally distributed whole blood glucose and plasma insulin data reported as mean and standard deviation for each group at all time points. Non-normally distributed serum cortisol and plasma ACTH data reported as median and interquartile range for each group at all time points. Significant increases from baseline for the intrasynovial group are denoted by the letter a (*p* < 0.05) and for the extrasynovial group by the letter b (*p* < 0.05) as determined by 2-way repeated measures ANOVA. No significant differences were found between groups.

Whole blood glucose values for the IS versus ES group are displayed in Figure 2A. Glucose values were increased from baseline at all time points for both groups (*p* < 0.05). There was no significant difference in glucose values between groups at any time point.

### 3.4 Insulin

Plasma insulin levels for the IS versus ES group are displayed in Figure 2B. Insulin levels for the IS group were increased from baseline at 10, 12, 16, 20, 24, and 60 h post-injection (*p* < 0.05). Insulin levels for the ES group were increased from baseline at 20, 24, 48, and 72 h post-injection (*p* < 0.05). There was no significant difference in insulin values between groups at any time point.

**Figure 3:**
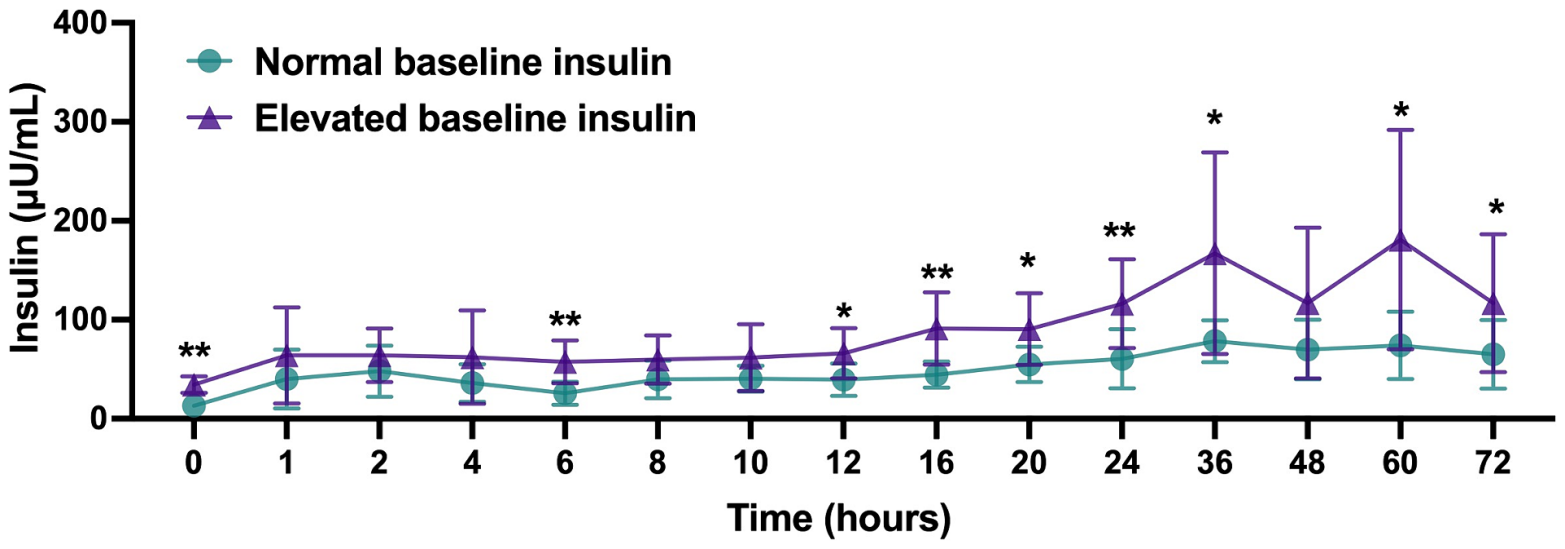
Plasma insulin (μU/mL) values following administration of 18 mg triamcinolone acetonide in horses with normal (<20 μU/mL, *n* = 9) or elevated (>20 μU/mL, *n* = 11) baseline insulin values. Data reported as mean and standard deviation for each group at all time points. Significant differences between groups are denoted by *(*p* < 0.05) or **(*p* < 0.01) as determined by 2-way repeated measures ANOVA.

**Figure 4:**
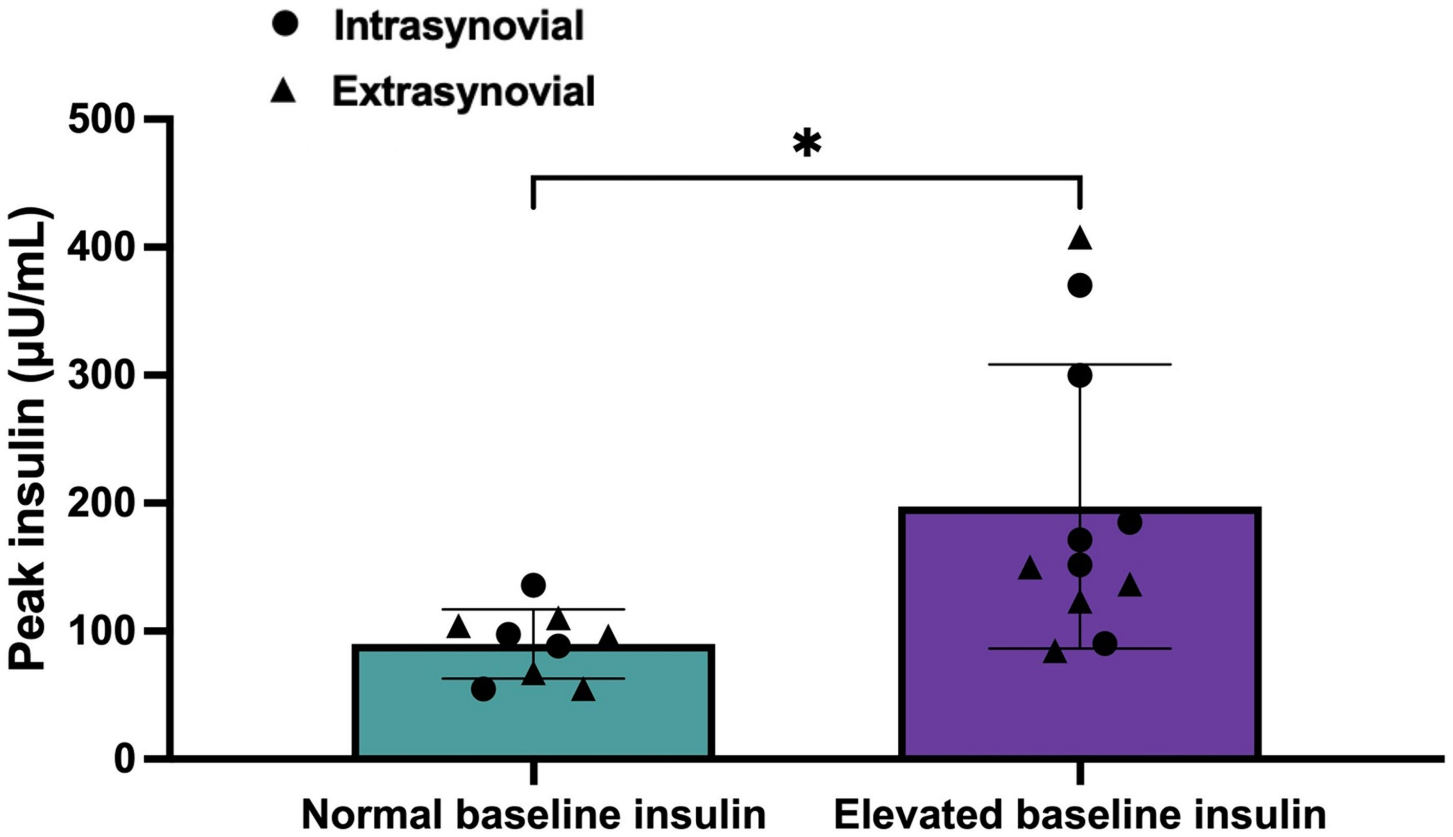
Peak plasma insulin (μU/mL) values following administration of 18 mg triamcinolone acetonide in horses with normal (<20 μU/mL, *n* = 9) or elevated (>20 μU/mL, *n* = 11) baseline insulin values. Peak values for each horse as well at the mean and standard deviation for each group are reported. Horses from the intrasynovial group are denoted by black circles and horses from the extrasynovial group are denoted by black triangles. *t*-test was used to determine significant difference as denoted by *(*p* < 0.05) between groups.

Plasma insulin values for horses with normal (<20 μU/mL) baseline values (*n* = 9; 4 from IS group and 5 from ES group) as compared to horses with elevated (>20 μU/mL) baseline values (*n* = 11; 6 from IS group and 5 from ES group) are displayed in Figure 3. Insulin levels for horses with elevated baseline insulin were higher at 0, 6, 12, 16, 20, 24, 36, 60, and 72 h post-injection compared to horses with normal baseline insulin (*p* < 0.05). Peak insulin values for horses in each group are displayed in Figure 4. Horses with elevated baseline insulin reached a peak insulin concentration of 197.5 ± 111.0 μU/mL which was higher than the peak insulin of 90.06 ± 26.92 μU/mL observed in the group with normal baseline insulin (*p* < 0.05).

Horses with elevated baseline insulin were 9 times more likely to reach peak insulin >100 uIU/mL post injections compared to horses with normal baseline insulin (OR = 9, 95% CI, 1.14–71).

### 3.5 Cortisol

Serum cortisol levels for the IS versus ES group are displayed in Figure 2C. Starting at 4 h post-injection and continuing until 72 h post-injection cortisol levels were decreased from baseline for both groups (*p* < 0.05). There was no significant difference in cortisol values between groups at any time point.

### 3.6 ACTH

Plasma ACTH levels for the IS versus ES group are displayed in Figure 2D. Starting at 4 h post-injection and continuing until 60 h post-injection ACTH levels were decreased from baseline for the IS group (*p* < 0.05). For the ES group, ACTH levels were decreased from baseline from 4 to 16 h post injection, and at 24, 36, 60, and 72 h post-injection (*p* < 0.05). There was no significant difference in ACTH values between groups at any time point.

## 4 Discussion

In this study we demonstrated differences in triamcinolone absorption between intra- and extrasynovial injection sites and added to the body of literature investigating the impact of corticosteroid administration on downstream metabolic parameters. This is the first study to evaluate drug absorption from the sacroiliac site, and the first to evaluate the impact of triamcinolone acetonide administration on ACTH levels.

Contrary to our hypothesis, plasma TA levels were significantly greater following intrasynovial injection than extrasynovial injection from 8–36 h post-injection. The peak plasma TA levels for the intrasynovial group (1.61 ng/mL) were similar to those previously reported by Soma et al. using a 0.04 mg/kg triamcinolone dose (0.94–2.5 ng/mL) (8). The peak TA levels for the extrasynovial group (0.70 ng/mL) were significantly lower than the intrasynovial group in this study, but higher than those reported for intramuscular administration (0.20–0.48 ng/mL) in the Soma study. This finding implies that the drug absorption properties of the sacroiliac joint injection site differ from both intrasynovial and intramuscular injection sites, which is reasonable given the nature of this extrasynovial injection close to the joint but also likely within the surrounding ligaments and muscle. A pharmacokinetic study evaluating absorption from extrasynovial injection sites would be beneficial in better understanding these findings.

Despite significant differences in triamcinolone absorption between groups, there was no significant difference in glucose, insulin, cortisol, or ACTH at any time point between groups and therefore the clinical relevance of this triamcinolone absorption finding is unclear. The degree of hyperglycemia seen in this study population was similar to that reported previously (9, 10). Glucocorticoids cause increased gluconeogenesis along with decreased tissue glucose uptake and relative insulin resistance, which is the likely mechanism for the hyperglycemia seen here (23, 24). Although the degree of hyperglycemia was mild from a clinical perspective, it may have contributed to subsequent hyperinsulinemia.

Horses in this study experienced significant hyperinsulinemia for up to 72 h post-injection. As sample collection was discontinued at the 72 h time point, the true duration of hyperinsulinemia for all horses could not be determined. The insulin response showed a high degree of individual variability with maximum insulin values ranging from 54.95 to 408.07 μU/mL. A large cohort study of ponies in England stratified ponies into low-, medium-, and high-risk groups for laminitis based on basal insulin levels. Ponies in the low-risk group (baseline insulin <21.6 μU/mL) had a 4 years cumulative incidence of laminitis of only 6%, while those in the high-risk group (baseline insulin >45.2 μU/mL) had an incidence of 69% (11). This modest degree of hyperinsulinemia was transiently present in all horses in the present study and persisted for at least 48 h in all but three horses. Sustained insulin levels of >208 μU/mL have been demonstrated to induce histopathologic evidence of laminitis in as little as 48 h (25). Three horses in this study achieved this degree of hyperinsulinemia, representing a group of horses that may be at increased risk for corticosteroid-associated laminitis. Although horses were not screened for endocrinopathies prior to inclusion in this study, all three of these horses did have elevated baseline insulin values (32.27 μU/mL, 47.70 μU/mL, 37.70 μU/mL). Despite this significant degree of hyperinsulinemia, no clinical signs of laminitis were observed in any horse. Radiographs or histopathologic evaluation of the laminae were not performed, but may have been able to detect laminitis that was not clinically evident.

The recent Boger et al. study evaluated the insulin and glucose response to the same dose of intrasynovial TA used presently, but only included horses with no evidence of insulin dysregulation as determined by an oral sugar test (10). Significant elevations in insulin were identified at 6, 24, and 48 h post-injection, but the mean peak insulin level was only 29 μU/mL as compared to the mean peak of 132.85 μU/mL found in our study population. The horses in the Boger study population had normal insulin response to an oral sugar test, while horses in our study population were not screened for insulin dysregulation prior to inclusion. In the subset of horses in our study population with elevated baseline insulin the mean peak insulin level was even higher at 197.5 μU/mL, while the mean peak insulin value for those with normal baseline insulin was only 90.06 μU/mL. Horses with elevated baseline insulin were nearly equally distributed between IS and ES groups and were 9 times more likely to reach a peak insulin of >100 μU/mL than those with normal baseline insulin. Baseline insulin is an insensitive measure of insulin dysregulation, so it is possible that a subset of horses with normal baseline insulin in our study population would have abnormal insulin responses to an oral sugar test. The presence of horses with insulin dysregulation undetected by baseline insulin abnormalities would explain the higher mean peak insulin value in our study population as compared to the Boger study population. Additionally, the use of an ELISA in our study versus a radioimmunoassay in the Boger study limits the ability to directly compare results. However, these findings argue that corticosteroids may induce more severe insulin dysregulation in horses with pre-existing baseline insulin dysregulation than those without, and that screening horses for insulin dysregulation may be an important step in mitigating risk of corticosteroid-associated laminitis. Further studies directly comparing the insulin response to corticosteroids in horses with diagnosed insulin dysregulation on the basis of an oral sugar test to those without are needed to confirm this finding.

Consistent with a previous study evaluating the impact of triamcinolone administration on endogenous hydrocortisone production, horses in this study experienced a significant decrease in cortisol levels from 4–72 h post-administration of triamcinolone. Additionally, a significant decrease in ACTH was present for the same time period. This is the first study to evaluate the ACTH response to triamcinolone administration, although previous studies have demonstrated ACTH suppression following administration of other corticosteroids (26, 27). These changes are indicative of suppression of the hypothalamic-pituitary-adrenal axis which may put patients at risk for secondary infections, and result in a clinical syndrome of ill-thrift, weight loss, and poor hair coat due to loss of normal cortisol functions (24). This risk should especially be considered in horses receiving repeated joint therapy with corticosteroids.

This study had several limitations, the first of which is that horses were unable to serve as their own controls due to the extended washout period that would be necessary before repeat assessment. Horses were also not screened for the presence of joint pathology, obesity, endocrinopathies, or laminitis prior to inclusion in the study, which may have affected drug absorption or the metabolic response to treatment. Additionally, horses in both groups were injected with amikacin combined with TA, and there was no TA only control. While there is no evidence in the current literature to suspect that amikacin could affect insulin levels or other metabolic parameters, it is unknown how this may have influenced study results. Horses were not evaluated for signs of laminitis with hoof testers or radiographs which may have allowed for detection of mild laminitis changes. A more prolonged sample collection period with more intensive monitoring may have been beneficial to determine the duration of metabolic changes and possible side effects that occur following triamcinolone administration. Finally, the use of an ELISA for insulin quantification limits the ability to directly compare the insulin values from our study population to others using the radioimmunoassay.

In conclusion, this study is the first to evaluate drug absorption from the sacroiliac site and demonstrated that systemic absorption of triamcinolone acetonide is greater from intrasynovial injection sites as compared to extrasynovial. The clinical relevance of this difference in absorption between sites is unclear as triamcinolone administration in horses in both groups resulted in hyperglycemia, hyperinsulinemia, and hypothalamic–pituitary–adrenal axis suppression up to 72 h post-injection. Hyperinsulinemia in some horses was profound and reached levels previously documented to increase risk for laminitis. There was a nearly equal distribution of horses with elevated baseline insulin between intrasynovial and extrasynovial groups and these horses had a significantly increased risk of developing marked hyperinsulinemia post-treatment. Further research is needed to determine if other corticosteroids and doses cause the same degree of metabolic derangements, and to determine the impact of these metabolic derangements on laminitis risk. Trends seen in this study indicate that screening for underlying insulin dysregulation may be an important tool in reducing risk of corticosteroid-associated laminitis, but additional studies are needed to confirm this finding.

## Data availability statement

The raw data supporting the conclusions of this article will be made available by the authors, without undue reservation.

## Ethics statement

The animal study was approved by North Carolina State University Institutional Animal Care and Use Committee (Protocol #22-115). The study was conducted in accordance with the local legislation and institutional requirements.

## Author contributions

KH: Conceptualization, Data curation, Formal analysis, Funding acquisition, Investigation, Methodology, Project administration, Resources, Supervision, Validation, Visualization, Writing – original draft, Writing – review & editing, Software. KD: Conceptualization, Data curation, Formal analysis, Funding acquisition, Investigation, Methodology, Resources, Supervision, Writing – original draft, Writing – review & editing. CH: Conceptualization, Data curation, Investigation, Methodology, Resources, Supervision, Writing – review & editing. HK: Data curation, Formal analysis, Methodology, Resources, Writing – review & editing. KM: Conceptualization, Formal analysis, Methodology, Writing – review & editing. LS: Conceptualization, Data curation, Formal analysis, Funding acquisition, Investigation, Methodology, Project administration, Resources, Supervision, Validation, Visualization, Writing – original draft, Writing – review & editing.
